# Immune modulation via adipose derived Mesenchymal Stem cells is driven by donor sex in vitro

**DOI:** 10.1038/s41598-021-91870-4

**Published:** 2021-06-14

**Authors:** Flyn Mckinnirey, Benjamin Herbert, Graham Vesey, Sharon McCracken

**Affiliations:** 1 Women and Babies Research, Perinatal Medicine, The University of Sydney, Faculty of Medicine and Health, and Northern, Sydney Local Health District Research (Kolling Institute), St Leonards, NSW 2065 Australia; 2 Sangui Bio Royal North Shore Hospital), St Leonards, NSW 2065 Australia; 3 Regeneus Ltd, 2 Paddington Street, Paddington, NSW 2021 Australia

**Keywords:** Mesenchymal stem cells, Stem-cell biotechnology, Stem-cell therapies, Stem-cell research

## Abstract

Mesenchymal stromal/stem cells (MSCs) are currently being used in clinical trials as proposed treatments for a large range of genetic, immunological, orthopaedic, cardiovascular, endocrine and neurological disorders. MSCs are potent anti-inflammatory mediators which are considered immune evasive and employ a large range of secreted vesicles to communicate and repair damaged tissue. Despite their prolific use in therapy, sex specific mechanism of action is rarely considered as a potential confounding factor for use. The purpose of this study was to examine the potency and functionality of both female and male adipose derived MSCs in order to gain further insights into donor selection. Methods MSC were expanded to passage 4, secretome was harvested and stored at − 80c. To assess potency MSC were also primed and assessed via functional immune assays, ELISA, multiplex and immunophenotyping. Results Female MSCs (fMSC), consistently suppressed Peripheral blood mononuclear cell (PBMC) proliferation significantly (*p* < 0.0001) more than male MSC (mMSC). In co-culture mPBMCs, showed 60.7 ± 15.6% suppression with fMSCs compared with 22.5 ± 13.6% suppression with mMSCs. Similarly, fPBMCs were suppressed by 67.9 ± 10.4% with fMSCs compared to 29.4 ± 9.3% with mMSCs. The enhanced immunosuppression of fMSCs was attributed to the production of higher concentrations of the anti-inflammatory mediators such as IDO1 (3301 pg/mL vs 1699 pg/mL) and perhaps others including IL-1RA (1025 pg/mL vs 701 pg/mL), PGE-2 (6142 pg/mL vs 2448 pg/mL) and prolonged expression of VCAM-1 post activation relative to mMSCs. In contrast, mMSCs produces more inflammatory G-CSF than fMSCs (806 pg/mL vs 503 pg/mL). Moreover, IDO1 expression was correlated to immune suppression and fMSCs, but not mMSCs induced downregulation of the IL-2 receptor and sustained expression of the early T cell activation marker, CD69 in PBMCs further highlighting the differences in immunomodulation potentials between the sexes. Conclusion In conclusion, our data shows that female MSC are more potent in vitro than their male counterparts. The inability of male MSC to match female MSC driven immunomodulation and to use the inflammatory microenvironment to their advantage is evident and is likely a red flag when using allogeneic male MSC as a therapeutic for disease states.

## Introduction

### Background

Mesenchymal stem/stromal cells (MSC) are a multi-potent, multifunctional cell type that are defined by their capability to proliferate, renew, differentiate and regenerate^1^. Tissue specific differences in MSC function and gene expression have been determined when comparing MSCs derived from adipose tissue, bone marrow, dental pulp, umbilical cord and more^2,3^ regardless of their origin, MSCs are recognised as immunomodulatory, angiogenic, anti-fibrotic, chondrogenic, and anti-bacterial^4^. The applications for MSC and their secretome within research and industry is broad due to their diverse functionality^5–7^ and with the expanding use of MSCs, like many other therapies, close consideration should be given to donor and recipient interaction.

The most well documented function of MSC is their immunosuppressive and immunomodulatory characteristics which, when administered as treatment have been shown to provide safe and acute relief and can potentially have long lasting effects. Clinical efficacy however, has been challenging with inconsistent results and uncommon proposed mechanism of action halting a widespread acknowledgement of the benefit of MSC therapy^8^.

Among others the International Society Cell and Gene Therapy have attempted to guide researchers and clinicians to a more harmonistic approach to MSC therapy by defining what an MSC should present^9–12^. However, the challenges associated with in vivo MSC efficacy potentially stem from a deeper set of characteristics such as donor and recipient age, secretion profile, homing capability, clearance rates, culture conditions, cryopreservation techniques and perhaps a rarely noted link to efficacy, donor and recipient sex^13–16^

### Immune modulation

MSC driven immunomodulation is a multi-pathway and multi-cellular strategy involving MSC immunogenicity and a synergy of secreted and surface communication molecules^17^. Immunogenicity or “immune-evasiveness” meaning MSCs lack immune system identifiers including Major Histocompatibility Complex II (MHC-II) and co-stimulatory molecules^18^, is key to MSC function. This characteristic allows MSCs to home to target tissue without immediate immune detection and mediating inflammation.

MSCs rely on co-ordinated cellular communication to alter inflammation. The molecules involved in MSC immunomodulation include messenger particles like extracellular vesicles^19^, soluble factors like cytokines and chemokines and also cell surface markers involved in homing and cell-to-cell contact which allow for more potent paracrine effects^20^. Many of these molecules are regulated by an inflammatory microenvironment stimulated by tissue injury and subsequent infiltrating immune cells which the MSCs are exposed to once administered^21^. Immune cells and subsequent secretion of proinflammatory factors like TNF-α, IL-1 and IFN-γ along with various other chemokines and free radicals^22,23^ then alter the MSC to a more potent cell type increasing the expression of immunomodulatory molecules such as Indoleamine 2,3, Dioxygenase (IDO), Prostaglandin E2 (PGE-2), Hepatocyte Growth Factor (HGF), Transforming Growth Factor (TGF-B1) and Interleukin-10 (IL-10)^24–26^ and upregulating immunomodulatory cell surface markers such as Intercellular adhesion molecule (iCAM), Vascular Adhesion molecule (VCAM), Programmed Death Ligand-1 (PD-L1) and Major and Minor Histocompatibility complexes (MHC) also known as Human Leukocyte antigen (HLA)^27^. These molecules alter immune cell differentiation, proliferation and maturation and shift the microenvironment from pro-inflammatory to anti-inflammatory^17,28,29^. Considering the cascade of events and the well-known disparities between the sexes, it seems ignorant to apply a one size fits all approach to MSC therapy.

### Sex considerations

#### Recipient

It has long been recognised that sexual dimorphisms exist and male and females show a sex specific susceptibility to certain diseases^30–32^. Fundamental differences in adipose tissue itself, a major source of MSC have been explored. Like other organs in the body, adipose tissue is receptive to a variety of factors which influence distribution and function and could affect the health and potency of MSCs derived from this tissue. Generally seen to be driven by sex hormones, oestrogens and androgens, this MSC starting material has shown major differences in receptor activity, metabolism, proliferation, fibrosis, gene expression and inflammation between male and females^33^.

The immune system is an extremely complex and responsive system which dictates almost every aspect of an animal’s ability to survive and function properly. Although sex disparities have been thoroughly researched, it has not been fully explored in biological therapies where sex matching may be advantageous. Females have more alert immune systems than males and better capabilities to produce antibodies which can make them more resistant to certain infections^34^. This has largely been attributed to oestrogen signalling which promotes inflammatory cytokines and Toll-like receptor (TLR) expression leading to increased activity of T cells^35^. The expression of specific TLRs differs between the sexes, with TLR3,7 and 9 expressed greater in females and TLR2 and 4 expressed higher in males indicating a fundamental disparity^34^. This alternate activation gives rise to differences in pathogen response and activation of downstream effector molecules^34,36,37^. The opposite is the case for males, where testosterone inhibits proinflammatory cytokine production, decreases TLR expression and subsequently decreases T cell activation by male Antigen Presenting Cells (APCs), resulting in lower rates of rejection of pathogens^34,35^.

#### Donor

The expansive capabilities of MSCs come with inherent risks and have posed considerable questions in the scientific community^15^ therefore, obtaining the most suitable starting material for the proposed indication and individual needs should be carefully considered.

This research focused on key MSC potency markers and subsequent immune modulation to determine if sex, not only of the starting material but recipient tissue had an effect on MSC efficacy. To do this we mimicked an inflammatory microenvironment using cytokines and Peripheral Blood Mononuclear cells (PBMCs) to assess MSC donor potency and probable efficacy.

## Materials and methods

### Ethics statement

Ethical approval for this study was provided by Northern Sydney Local Health District Human Research Ethics Committee for the use of Mesenchymal stem cells in the analysis of sex differences in Osteoarthritis (Regis 2019_ETH00501). All research was performed in accordance with relevant guidelines and regulations^38^.

### Media preparation

This research used multiple media types including; *Stromal Vascular Fraction (SVF) growth media* [used for Passage 0 MSCs] Alpha Modified Essential Medium (αMEM) (Lonza, Australia) with 10% Platelet lysate (PLT) (Cook Regentec, United States) and 1% Antibiotic–Antimycotic (ABAM -ThermoFisher, Australia), *MSC growth media* [used for Passage 1 to Passage 4 MSCs] αMEM with 10% PLT, *Priming Media* [used to activate MSCs] αMEM with 10% PLT supplemented with 10 ng/mL Tumour Necrosis Factor (TNF-α) (Stem cell technologies, Australia) and 100 ng/mL Interferon Gamma (IFN-γ) (Stem cell technologies, Australia) and *PBMC media* Roswell Park Memorial Institute Media (RPMI)(Sigma, Australia), 1% ABAM and 10% Foetal Bovine Serum (FBS).

### MSC preparation

#### Processing adipose tissue

MSCs were isolated from Stromal Vascular Fraction (SVF) after autologous stem cell therapy by Stem Cell company, Regeneus Ltd. Stromal vascular fraction is a heterogenous population of cells obtained from adipose tissue which contains a small population of MSCs. All patients gave written informed consent for cells to be used in future research.

SVF was isolated according to Zuk et al.^39^. Briefly, lipoaspirate samples were digested using 0.05% wt./vol collagenase (Sigma, Australia), washed using Dulbecco’s Modified Eagle Medium (ThermoFisher, Australia) to remove collagenase and pelleted via centrifugation. The resultant SVF were counted and viability determined via FACSCalibur (data not shown). SVF was then cryopreserved in Cryostat (CS10) (Stem cell technologies, Australia) in LN_2_ at a concentration of ~ 1 × 10^7^ cells/mL.

#### Cell expansion

Adipose derived MSCs were isolated from stored SVF samples. Frozen SVF was thawed at 37 °C in a water bath for 2 min and cultured at 12,000 cells/cm^2^ *in SVF growth media* in cell culture treated flasks. When cells reached ~ 90% confluence cells were harvested and counted. Further MSC expansion was achieved in *MSC growth media* at 37 °C in 5% CO_2_. Once MSCs had been passaged four times (P4) they were upscaled into a 2-layer cell factory (ThermoFisher, Australia) to obtain ~ 1 × 10^8^ cells per donor. MSC secretome (MSC-S) was harvested by decanting, centrifuged and frozen at − 80 °C. MSC were harvested when confluence reached 90–100%. Cells were counted and viability determined using the FACSCalibur after each harvest and stored at 2 × 10^6^ cells per aliquots in CS10 (Stem cell technologies, Australia) at − 80 °C then transferred to LN_2_ 12 h later.

#### FACS cell count and viability

To determine cell count and viability (CCV), cells were stained with Propidium Iodide (PI) (Sigma, Australia) and SYTO 11 (ThermoFisher, Australia) in Trucount tubes (BD Biosciences, Australia) and analysed via FACSCalibur.

### Cell characterisation

#### Donors

Age and health status of all MSC donors was seen to be matched for both sexes.. Four female MSC donors average age 45 and four male MSC donors average age 46 (supplementary table S1) were cultured to passage 4 using *MSC growth media* and assessed for successful population doubling (> 2 per passage), protein analysis, and immunophenotyping. They were further exposed to inflammatory cytokines (MSC priming media) and Peripheral Blood Mononuclear cells. Three Male and three female PBMCs donors aged between 41 and 61 years old were purchased from Lonza (United States), thawed, washed and aliquoted into 5 × 10^6^ cells/vial in RPMI, 10% FBS, 10% DMSO for use in subsequent PBMC assays.

#### Morphology

Cells were monitored at each passage via microscopic imaging (Dino-lite, Australia).

#### Phenotyping

To determine the cell phenotype, MSC or PBMC aliquots (1 × 10^5^/test) were thawed, washed in PBS (Life Technologies, Australia) and subsequently washed with 1.0 mL Flow cytometry Buffer (ThermoFisher, Australia) by centrifuging at room temperature for 5 min at 500 g. Wash supernatant was discarded and 5 µL of appropriate conjugated flow cytometry Ab was added to the tube and incubated at 4 °C for 30 min. Cells were then washed and centrifuged twice, fixed using 200 µL BD FACS™ Lysing Solution (BD Bioscience, Australia) and stored at 4 °C overnight. Cells were run on a FACSCalibur and analysed using CellQuest.

#### Antibodies used for phenotyping

For Adipose derived MSC characterisation cells were labelled with CD90—Fluorescein isothiocyanate (FITC)(Thy-1), CD105 FITC (Endoglin), CD73 FITC (lymphocyte-vascular adhesion protein 2/ 5′-nucleotidase/NT5E), CD13 FITC, CD29 FITC, CD44 FITC, CD34 FITC (hematopoietic stem cells and endothelial cells markers), CD271/NGFR FITC, Human Leukocyte Antigen (HLA)-DR FITC, CD45—(leukocyte marker) phycoerythrin (PE), CD19 PE, CD106 PE (VCAM), CD11b PE (integrin α M), CD166 PE, CD31 PE (R&D systems). MSC were also labelled with IgG1K antibodies against VCAM, iCAM (CD54 PE), PD-L1 (CD274 PE), HLA-ABC PE, HLA-G PE, HLA-E PE and IgG2bK antibody HLA-DR FITC (R&D systems, United States) to assess homing capabilities after priming. Cell surface markers for PBMCs assessed T cell markers CD3 PE, T _helper_ CD4 FITC, T _cytotoxic_ CD8 PE, B cell CD19 PE, CD25 PE and early activation marker CD69 FITC antibodies (R&D systems, United States). All samples were run on FACSCalibur and analysed using CellQuest.

### MSC-S characterisation

To assess the characteristics of donor MSC secreted protein, MSC-S was assessed under normal culture conditions and after 24 h priming with inflammatory cytokines. Analytes were measured and data was pooled using the Bio-plex Pro Human Cytokine 27-plex assay (IFN-y, IL-1b, IL-5, IL-9, IL-12, IL-15, 1L-17, TNF-α, IL-1ra, IL-4, IL-10, IL-13, Eotaxin, MCP-1, MIP-1α, MIP-1β, RANTES, FGFbasic, G-CSF, GM-CSF, IL-17, IP-10, PDGF-b, VEGF-a, IL-2, IL-6) (Bio-Rad, Australia), PGE-2 ELISA (ABCAM, Australia) and Custom ProcartaPlex Multiplex immunoassays (Thermo Scientific, Australia) (G-CSF, IDO, IFN-y, IL-1Ra, IL-6, IL-8, IP-10, MCP-1, TNF-α, VEGF-A, IL-2, IL-10, HGF, TGF-β1). Bioplex and ProcartaPlex are both Luminex based multiplex assays that utilise magnetic beads with antibodies directed against distinct analytes in the one assay, enabling measurement of many analytes at one time. Assays were performed as per manufactures instruction. Briefly, MSC-S was defrosted and spun at 10,000 g for 2 min, spun sample supernatant was bound, detection Ab conjugated with Streptavidin-PE were added and read on a MAGPIX 200 in the case of ProcartaPlex or Bioplex 200 for Bioplex.

### MSC priming assay

To assess functionality, MSCs were primed by exposure to inflammatory cytokines and assessed for known functional markers via FACS. ELISA and ProcartaPlex were used for assessment of secreted molecules and immune modulation was analysed via *a PBMC assay*.

#### MSC priming

MSC were thawed at 37 °C in a water bath for 2 min and seeded into 0.4 µm transwell plates (Corning) at 12,000 cells/cm^2^ in 1.0 mL *MSC culture media*. MSCs were allowed to recover for 2 days at 37 °C in 5% CO_2_ or until they reached 50% confluence (48–72 h). At 50% confluence, MSCs were primed by adding *Priming Media* to sample wells and incubated. MSCs and MSC-S were then either (1) harvested and frozen to assess MSC immunomodulatory characteristics after the 24 h incubation, (2) media changed to fresh *MSC culture media* and incubated for a further 24 h to analyse any sustained expression of immunomodulatory markers once removed from inflammation, harvested and frozen or (3) were utilised in the PBMC proliferation assay to assess their ability to suppress PBMC. Control MSC wells were unprimed.

### MSC co-culture

#### MSC priming

MSC mediated immunosuppression was analysed using a PBMC proliferation assay. Primed MSCs were seeded in the bottom chamber of a Transwell system (Corning, Australia) with 0.4uM pore size as to only allow soluble factors to pass and exposed to stained and activated PBMC.

#### PBMC staining and activation

PBMC aliquots were thawed and cultured overnight in PBMC media. Cells were then harvested and counted for CCV and stained with 2.5 µM CellTrace™ Carboxyfluorescein Diacetate Succinimidyl Ester (CFSE) (ThermoFisher, Australia) for 20 min at Room temperature in the dark. Cells were then quenched for excess stain by adding 15.0 mL *PBMC media* and incubated at room temperature for a further 10 min. PBMCs were then centrifuged for 7 min at 400 g and resuspended in 500uL prewarmed *PBMC media* prior to stimulation. PBMC were then induced to proliferate using the T cell Activation/Expansion kit (Miltenyi, Australia) which consists of Anti-Biotin MACSiBead Particles and biotinylated antibodies against human CD2, CD3, and CD28. Briefly, 2 × 10^6^ PBMC/assay were exposed to T-cell activation/expansion kit in *PBMC media* prior to co-culturing with primed MSCs. PBMC’s (~ 2 × 10^5^ cells) were added to the top layer of a transwell culture system with pre-primed MSCs (~ 5 × 104 cells) in the bottom chamber. The transwells were cultured in PBMC *media*. Untreated PBMCs were used as non-activated controls. Proliferation was assessed using FACScan and further analysis was performed using Flow logic.

#### PBMC proliferation

PBMC proliferation was assessed via FACSCalibur Flow cytometry. CFSE fluorescence was determined using dot plot and histogram analysis of dye dilution of PBMC generations. CFSE fluorescence of PBMC was compared to the untreated control. Untreated PBMCs show a high Mean Florescence intensity(MFI) reading and activated PBMCs show a low MFI reading. The MFI is measured as the mean intensity level of the CFSE dye, proliferating cells will show a lighter mean CFSE intensity (lower MFI). When primed MSC are included in co-culture with activated PBMCs the MFI of the PBMC was expected to be greater than untreated and less than activated control MFI readings and suppression was presented as a percentage according to the following equation.

#### Suppression rate

$$\mathrm{\% }Suppression=\frac{\mathrm{Co}-\mathrm{culture CFSE }(\mathrm{MFI})}{(\mathrm{Untreated PBMC CFSE }(\mathrm{MFI})-\mathrm{Activated PBMC CFSE }(\mathrm{MFI}))}*100$$

### Blocking IDO1

To test the effect of IDO1 on PBMC suppression we added the IDO inhibitor Epacadostat (EPA) (Selleck chemicals, United States) to the PBMC suppression assay. EPA was added at increasing concentrations at the time of priming and the effect on fMSC mediated immune suppression was assessed. Effective IDO1 suppression was demonstrated using the ProcartaPlex (ThermoFisher, Australia).

### Statistical analysis

Data are presented as mean ± standard error of the mean. Statistical significance was determined without correction for multiple comparisons using the Mann Whitney test, with *p* < 0.05 were considered as statistically significant and significance level is represented by * = * p* < 0.05, ** = * p* < 0.01, ***p < 0.001.

## Results

### Male and female MSCs have similar growth and phenotypical characteristics

**Figure 1:**
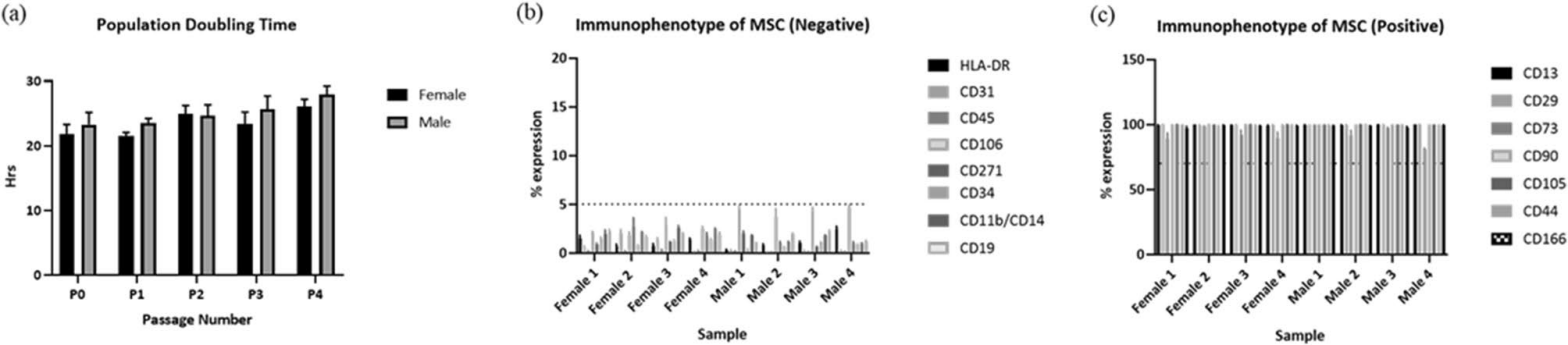
(**a**) Both male and female MSCs showed similar growth population doubling times (h), (**b**) did not express HLA-DR, CD31, CD45, CD106, CD271, CD34, CD11b/CD14 and CD19 and (**c**) were positive for CD13, CD29, CD73, CD90, CD105, CD44 and CDCD166.

MSC doubling per day and total doublings showed no significant differences between the donors up to passage 4 (Fig. 1a) and all MSCs showed a fibroblastic like morphology (supplementary figure S1).

Additionally, male and female showed cell surface expression patterns consistent with MSC characterisation^9,11^ negative for CD45, CD34, CD11b, HLA-DR and CD19 and Positive (≥ 70%) for classical MSC markers CD90, CD105, CD73. In addition, in an extended MSC characterisation panel all MSC were negative (≤ 5%) for CD31 and CD271 and positive for CD166, CD44, CD13 and CD29 and negative (≤ 5%) for CD31 and CD271 via FACS (Fig. 1b,c).

### Female MSCs have greater immunosuppressive properties than male MSCs

**Figure 2:**
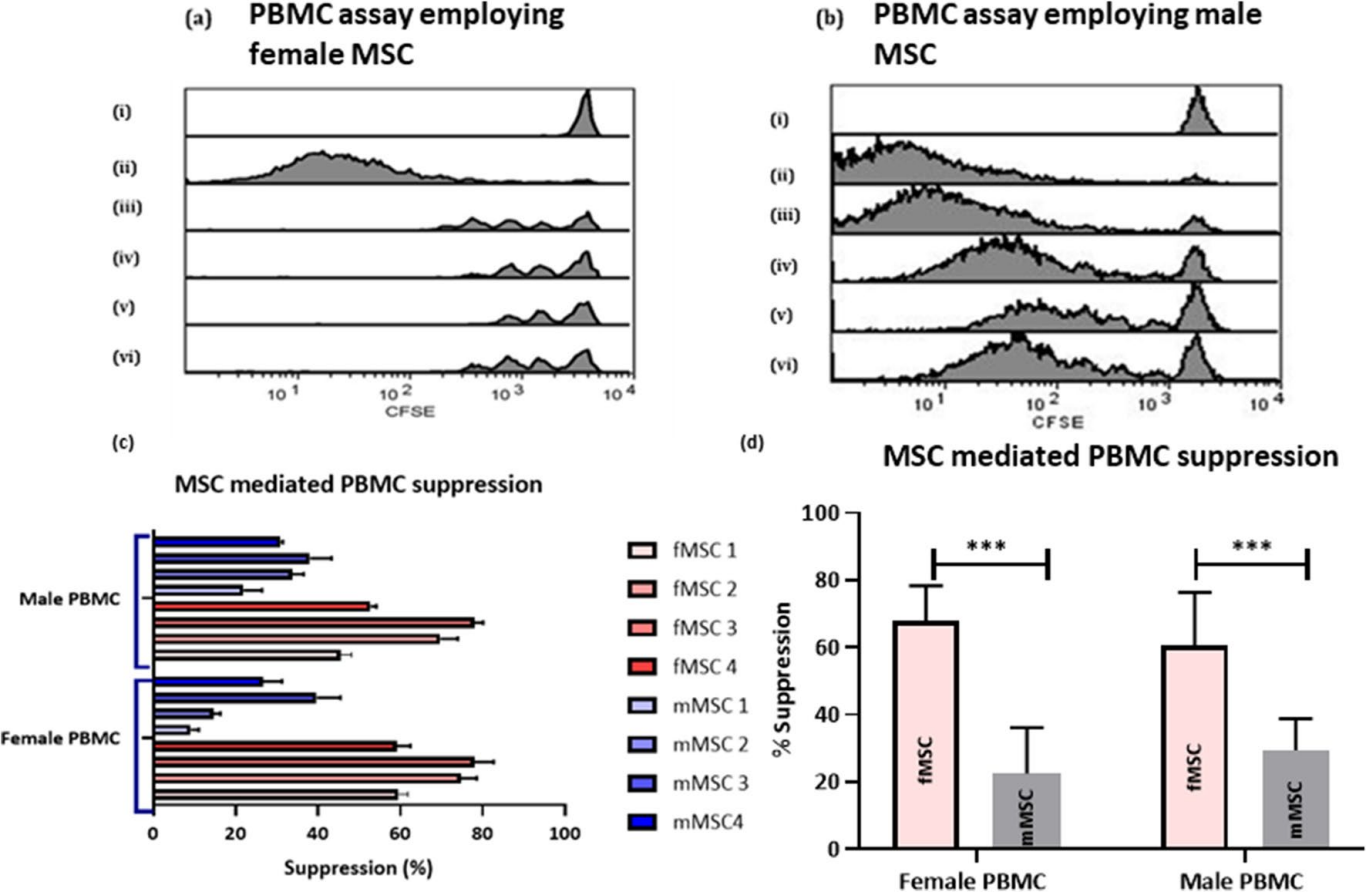
Example of FACS analysis of the PBMC proliferation assay using the FL-1 channel (**a**) PBMC proliferation assay using fMSC (i) control unstimulated fPBMC, (ii) control activated fPBMC, (iii) activated fPBMC + primedc fMSC donor 1, (iv) activated fPBMC + primed fMSC donor 2, (v) activated fPBMC + primed fMSC donor 3, (vi) activated fPBMC + primed fMSC donor 4 compared to (**b**) PBMC assay using mMSC (i) control unstimulated fPBMC, (ii) control activated fPBMC, (iii) activated fPBMC + primed mMSC donor 1, (iv) activated fPBMC + primed mMSC donor 2, (v) activated fPBMC + primed mMSC donor 3, (vi) activated fPBMC + primed mMSC donor 4. (**c**) Individual MSC mediated immune suppression of both male and female PBMC expressed as a percentage indicatiing the suppression rate and (**d**)suppression (mean ± SEM) of fPBMC (n = 3) and mPBMC (n = 3) against both fMSC (n = 4) and mMSC (n = 4).

To test for difference in the immunosuppressive properties of MSCs derived from males (mMSC) and females (fMSC), MSCs were used to alter PBMC proliferation. Isolated male and female PBMC were stained with CellTrace CFSE and subsequently stimulated with MACSiBead anti-CD2, anti-CD3, anti-CD28 microparticles. PBMC from both male and females were cultured with primed male and female MSCs and were analysed on FACSCalibur for immune suppression. Figure 2 shows representative histograms showing the effect of fMSC (Fig. 2a) and mMSC (Fig. 2b) on PBMCs. The greater the loss of fluorescence, the greater the amount of proliferation. The suppression of PBMCs proliferation is represented by a shift in fluorescent intensity toward the unstimulated control. All fMSCs showed a more pronounced shift toward unstimulated controls when combined with PBMCs relative to mMSCs (Fig. 2a,b). Moreover, fMSC donors consistently presented a higher suppression rate than mMSC (Fig. 2c). The suppression rates were significantly higher (*p* < 0.0001) when fMSC were used to suppress both male and female PBMC (60.7 ± 15.6 and 67.9 ± 10.4), relative to mMSC (22.5 ± 13.6 and 29.4 ± 9.3) (Fig. 2d). This data suggests increased MSC mediated suppression of PBMCs by fMSC compared to mMSC is entirely due to the competency of fMSC.

### Male and female MSCs respond similarly to inflammatory mediators

In addition to T-cell inhibition via paracrine mediators, it has long been recognised that cell surface markers including iCAM-1, VCAM-1 and PD-L1 play essential roles in MSC potency through their role in mediating cell-to cell contact^40–44^. Since primed fMSC have been shown to have a greater immune suppression than primed mMSC, this prompted us to ask whether the MSC response to inflammation was sex specific. To test this, we assessed the expression of iCAM-1, VCAM-1 and PD-L1 together with Interferon Gamma Receptor 1 (IFGR1) HLA-DR, HLA-ABC, HLA-G and HLA-E.

**Figure 3:**
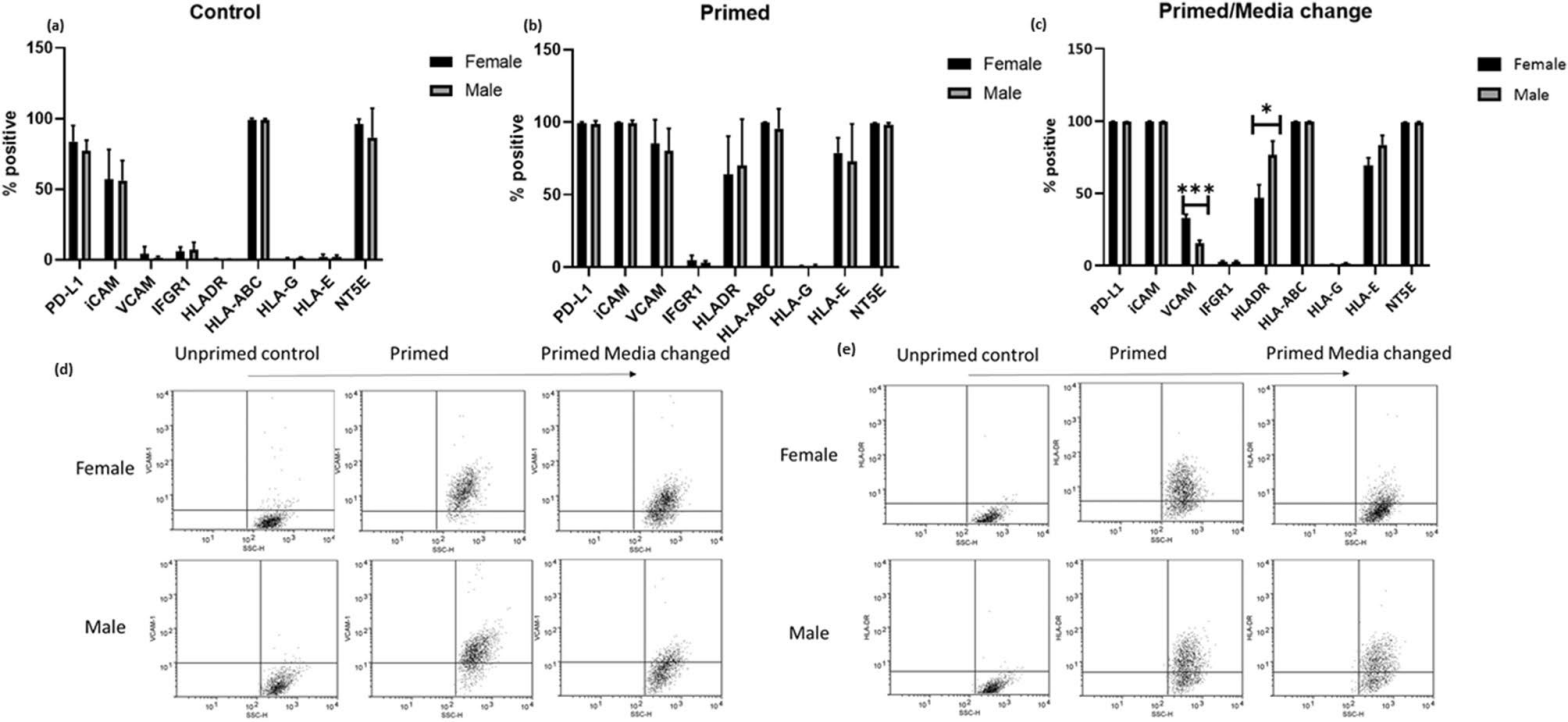
MSC surface marker expression (mean ± SEM) of (a) unprimed (no addition of IFN-γ/TNF-α) (**b**) pimed (100 ng/mL IFN-γ, 10 ng/mL TNF-α). Both fMSC and mMSC show similar cell surface characteristics when functional markers PD-L1, iCAM, VCAM, IFGR1, NT52 and HLA markers DR, ABC, G were analysed. (**c**) Cell surface markers were analysed 24 h post priming. (**d**,**e**) Dot plots for significance differences (*p* > 0.05) after media change for (**d**) VCAM and (**e**) HLA-DR (*p < 0.05, **p < 0.01, ***p < 0.001).

When MSC were *not* exposed (unprimed control) to inflammatory cytokines, there was no difference in the level of expression of iCAM, NT5E, HLA-ABC, PD-L1, HLA -DR, HLA-G, HLA-E or VCAM-1 (Fig. 3a).

When MSC were primed by exposing them to inflammatory cytokines, IFN-γ and TNF-α, (Fig. 3b) all donors upregulated VCAM-1 (fMSC 85.5 ± 16, mMSC 80.27 ± 15.4), iCAM-1 (fMSC 99.77 ± 0.2, mMSC 99.14 ± 2.1), PD-L1 (fMSC 99.3 ± 0.85, mMSC 98.8 ± 2.05), HLA-DR (fMSC 64.1 ± 26.2, mMSC 70.1 ± 32), HLA-E (fMSC 78.8 ± 10.4, mMSC 73.04 ± 25.7) and NT5E (fMSC 95.9% ± 3.8. mMSC 93.1 ± 3.6). In contrast, there was no effect on the expression level of HLA-G and IFNGR1. HLA-G remains virtually undetected (< 5%) across all samples tested.

This indicates that all MSC donors irrespective of sex, possess similar ability to regulate cell surface markers when presented with an inflammatory microenvironment.

### Post primed female MSCs sustain VCAM-1 while male MSCs sustain HLA-DR

Since there was no difference in the induction of cell surface receptor expression between male and female MSC, this prompted us to investigate how mMSC and fMSC respond post inflammation. MSCs were primed for 24 h and cultured for a further 24 h in *MSC culture media*. MSCs were assessed for immunoregulatory cell surface markers. Figure 3c–e shows fMSC sustain the presence of VCAM-1 (*p* = 0.002) more so than mMSC, while mMSC sustain HLA-DR expression more than fMSC (*p* = 0.04). There was no significant difference in the expression of PD-L1, iCAM, IFNGR1, HLA-ABC, HLA-G, HLA-E and NT5E 24 h post priming.

### Male and female MSCs selectively regulate CD8, CD25 and CD69 expression in activated PBMCs

It is well known that MSC interaction with CD4^+^ T cells is imperative to immunomodulation. MSCs alter T-cell phenotypes from an inflammatory to anti-inflammatory phenotype^45,46^. To investigate whether inherent differences exist between male and female PBMCs and/or how MSCs interact with PBMCs and alter their phenotype, we assessed cell surface markers of PBMCs alone (Resting and Activated) and after 6 days in co-culture with primed MSCs.

**Figure 4:**
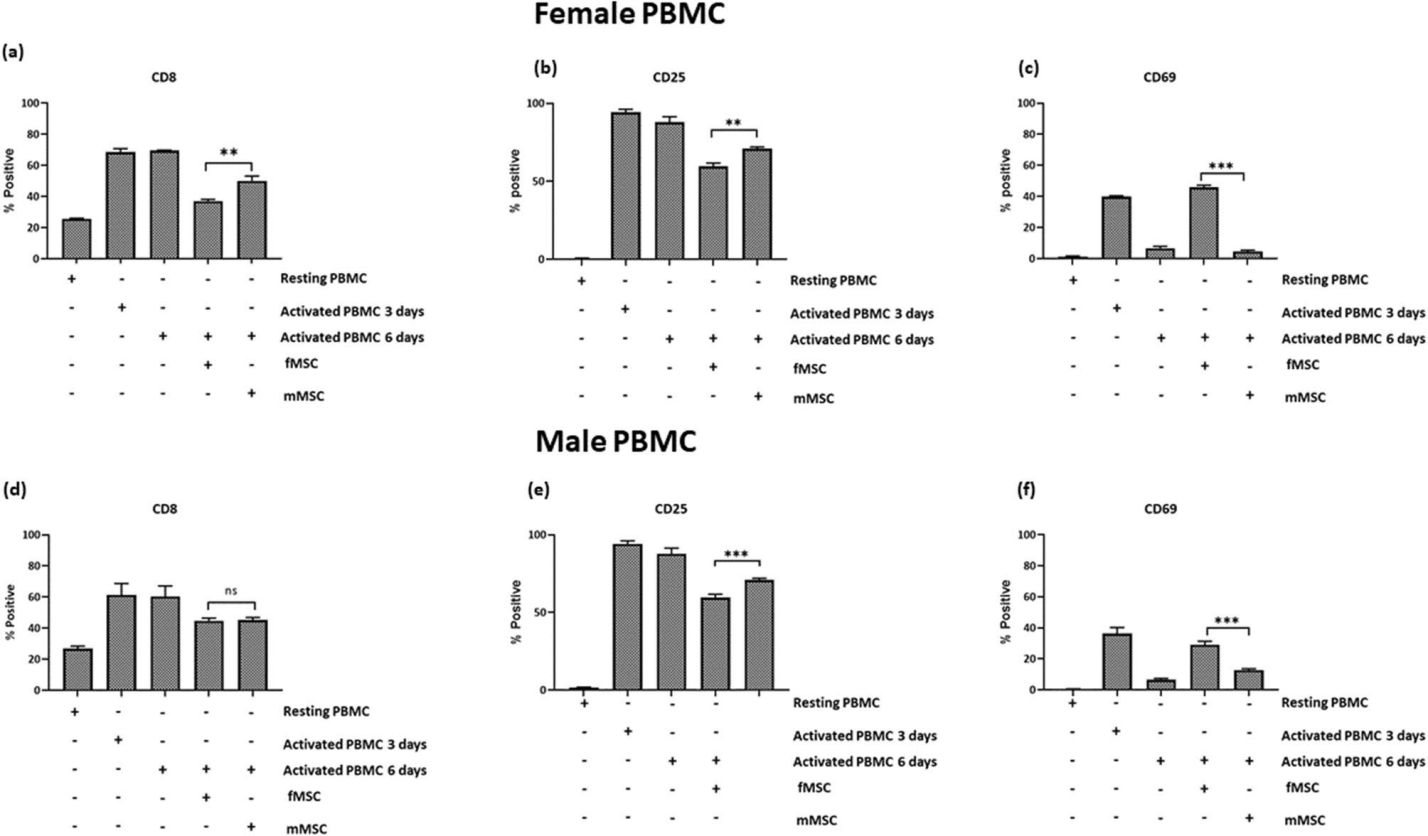
Female PBMC (resting, activated for 3 days, activated for 6 days, activated for 6 days and co-cultured with fMSC or mMSC) and analysed for the expression of (**a**) CD8, (**b**) CD25 and (**c**) CD69.Male PBMC (resting, activated for 3 days, activated for 6 days, activated for 6 days and co-cultured with fMSC or mMSC) were also analysed for the expression of (**d**) CD8, (**e**) CD25 and (**f**) CD69 (*p < 0.05, **p < 0.01, ***p < 0.001).

Analysis of the lymphocyte population showed no differences in the level of expression of CD3, CD4, CD8, IL-2 receptor CD25 or the early activation marker CD69 between male and female PBMCs when resting, activated with MACSiBead microparticles for 3 days (early activation) or after 6 days stimulation (supplementary Figure S3). CD8 and CD25 were upregulated in response to MACSiBead microparticle stimulation. CD8 and CD25 were increased after 3 days and remained high at day 6 (Fig. 4). In contrast, CD69 expression showed an early activation peak which declined by day 6 (resting 0.8%, 3 days activated 38.1% and 6 days activated 6.7%, Fig. 4).There was no change to CD19^+^ B cell numbers in response to MACSiBead microparticles (data not shown).

When activated PBMC were co-cultured with either mMSC and fMSC for 6 days , the expression of CD8 (Fig. 4a,d) and CD25 (Fig. 4b,e) was downregulated relative to activated PBMC without MSC. The sex of the MSCs dictated the level of suppression, mMSC were less effective at downregulating CD8 in female and CD25 in male and female PBMCs than fMSC (*p* < 0.05). More over, in the presence of fMSCs, but not mMSCs, CD69 expression in PBMC from both sexes was sustained for 6 days (Fig. 4c,f), indicating an MSC sex related difference in mediating PBMC cell surface marker regulation.

### Primed female MSCs produce higher levels of IDO, PGE-2 and IL-1RA but lower levels of G-CSF than male MSCs

To further examine the immunomodulatory capabilities of male and female MSC, we compared the secretion characteristics of both sexes post culture.

#### Positive analytes

There were no significant differences for Unprimed MSC-S from all donors for MCP-1, TGF-B/LAP, IL-8, IL-6, VEGF-A, Eotaxin, RANTES (supplementary Figure S3).

#### Induced analytes

**Figure 5:**
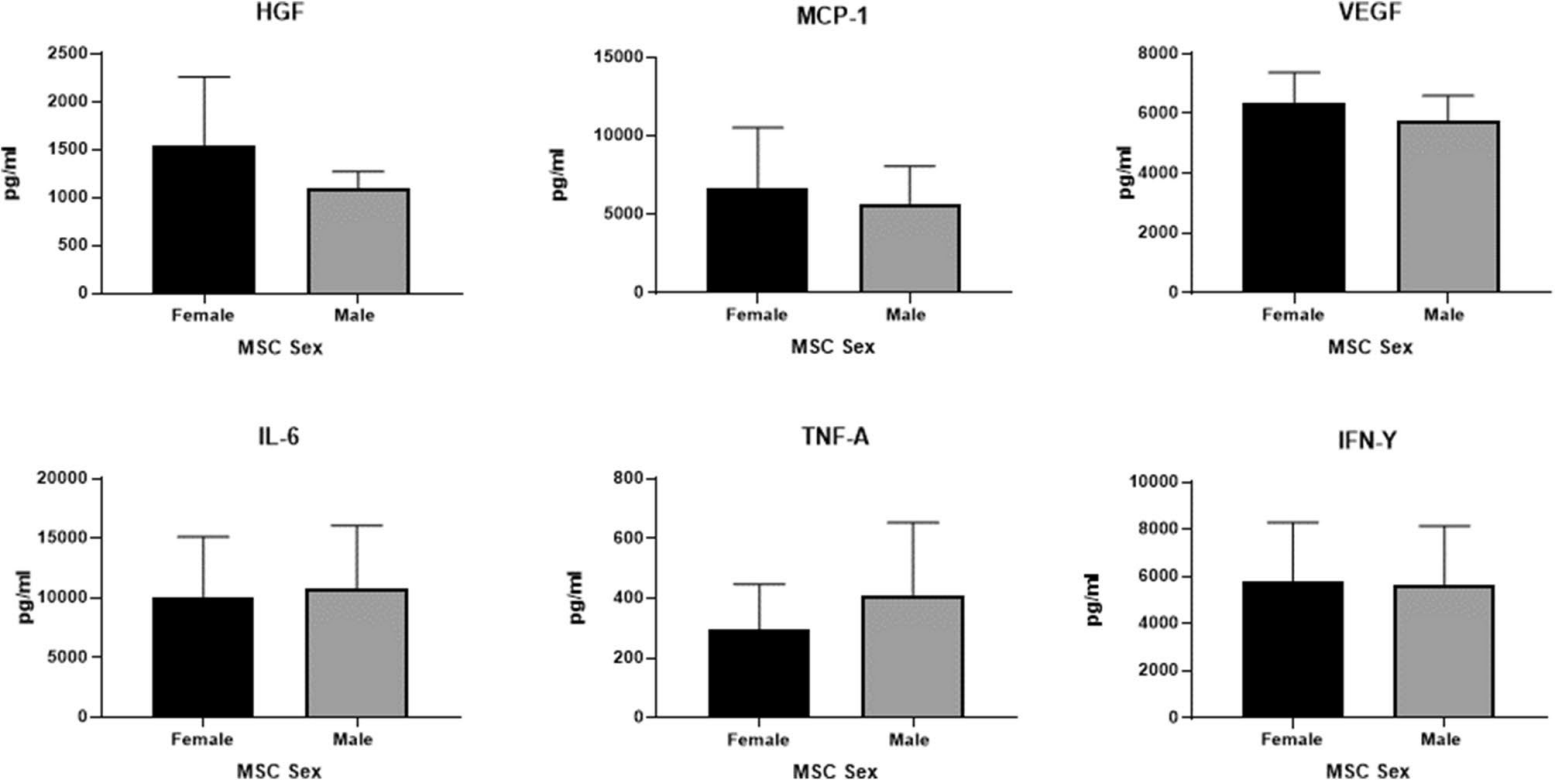
Analytes measured in the secretome of male and female MSCs primed for 24 h using Procartaplex (**a**) Hepatocyte growth factor (HGF), (**b**) Monocyte chemoattractant-1 (MCP-1), (**c**) vascular endothelial growth factor (VEGF-A), (**d**) interleukin-6 (IL-6), (**e**) tumor necrosis factor alpha (TNF-α) and interferon gamma (IFN-γ).

**Figure 6:**
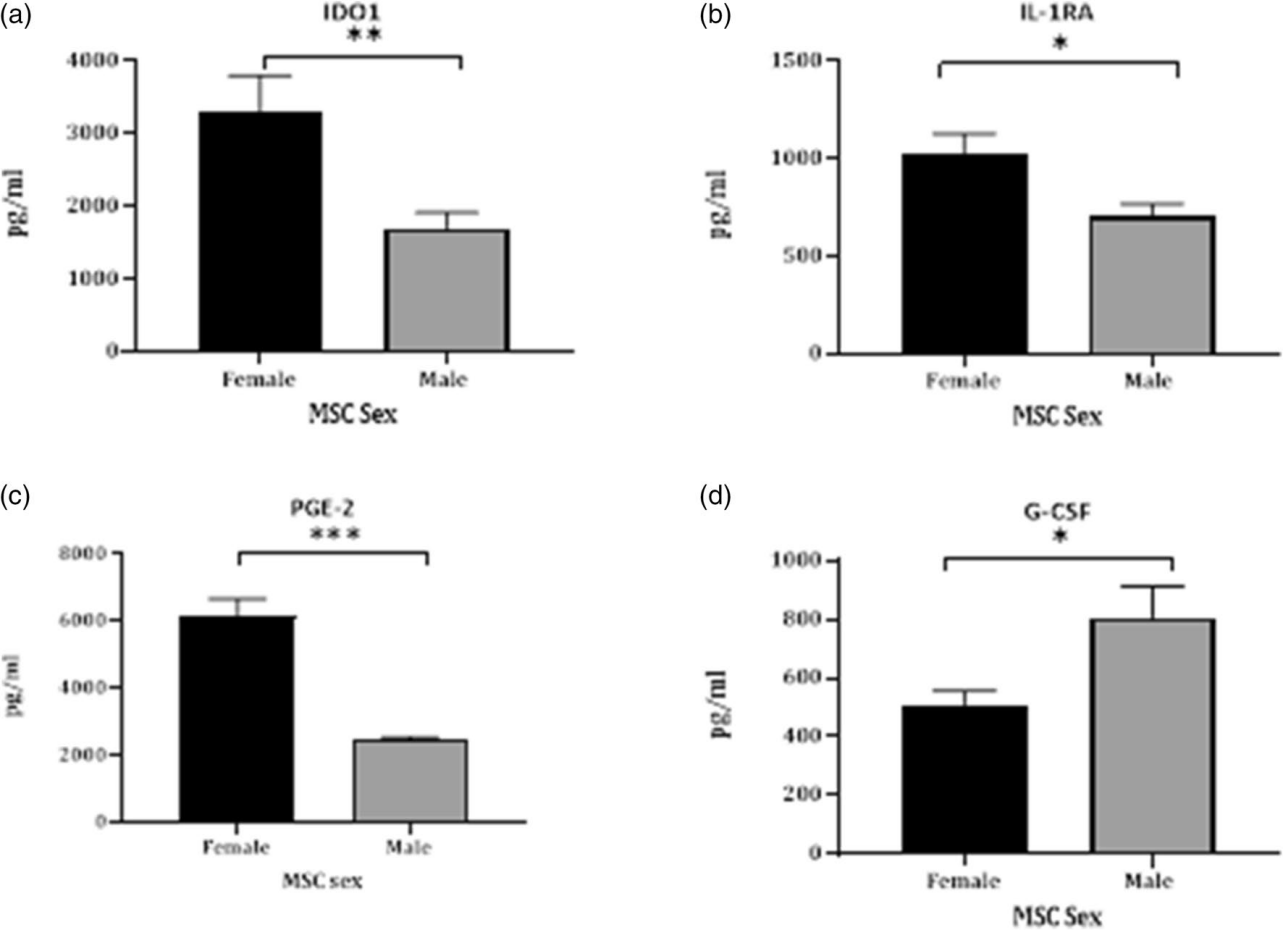
Analysis of secreted analytes from primed MSCs using the ProcartaPlex. (**a**) IDO1, (**b**) IL-1RA and (**c**) PGE-2 and whereas mMSC secrete higher levels of (**d**) G-CSF when primed with TNF-α and IFN-γ (*p < 0.05, **p < 0.01, ***p < 0.001).

To gain further insight into the paracrine mechanisms of MSC immunomodulation, we also harvested the MSC-S 24 h post priming and assessed protein expression. MSC-S from all donor’s post priming showed increased expression of MCP-1, TGF-B/LAP, IL-8, IL-6, VEGF-A, HGF, IP-10, MIP-1α and MIP-1β (data not shown). There was no significant difference in the level of HGF, MCP-1, VEGF-A, IL-6, TNF-α or IFN-y in the secretome from male and female MSCs (Fig. 5). In contrast, MSCs from all female donors secreted higher concentrations of IDO1 than their male counterparts (Fig. 6a 3301 pg/mL vs 1699 pg/mL respectively). Female activated MSC also showed increased expression of PGE-2 (Fig. 6c fMSC 6142 pg/mL vs mMSC 2448 pg/mL) and the “IL-1 inhibitor”, IL-1RA (Fig. 6b fMSC 1025 pg/mL vs mMSC 701 pg/mL) whereas the opposite was seen for G-CSF (Fig. 6d fMSC 503 pg/mL vs mMSC 806 pg/mL). Analytes that were assessed but not detectable are indicated in the Supplementary section.

### Inhibition of IDO function completely ablates the suppressive function of female MSCs

IDO1 is responsible for the catabolism of Tryptophan, which is an essential part of T-cell activation and proliferation. Our data shows fMSCs secrete greater quantities of IDO1 than mMSCs. To determine whether IDO contributed to the efficacy of fMSCs, IDO was inhibited from fMSCs using Epacadostat/INCB24360 (EPA) a known inhibitor of IDO1^47^. CFSE stained female PBMC were co-cultured with fMSC with and without EPA treatment and the level of suppression determined.

**Figure 7:**
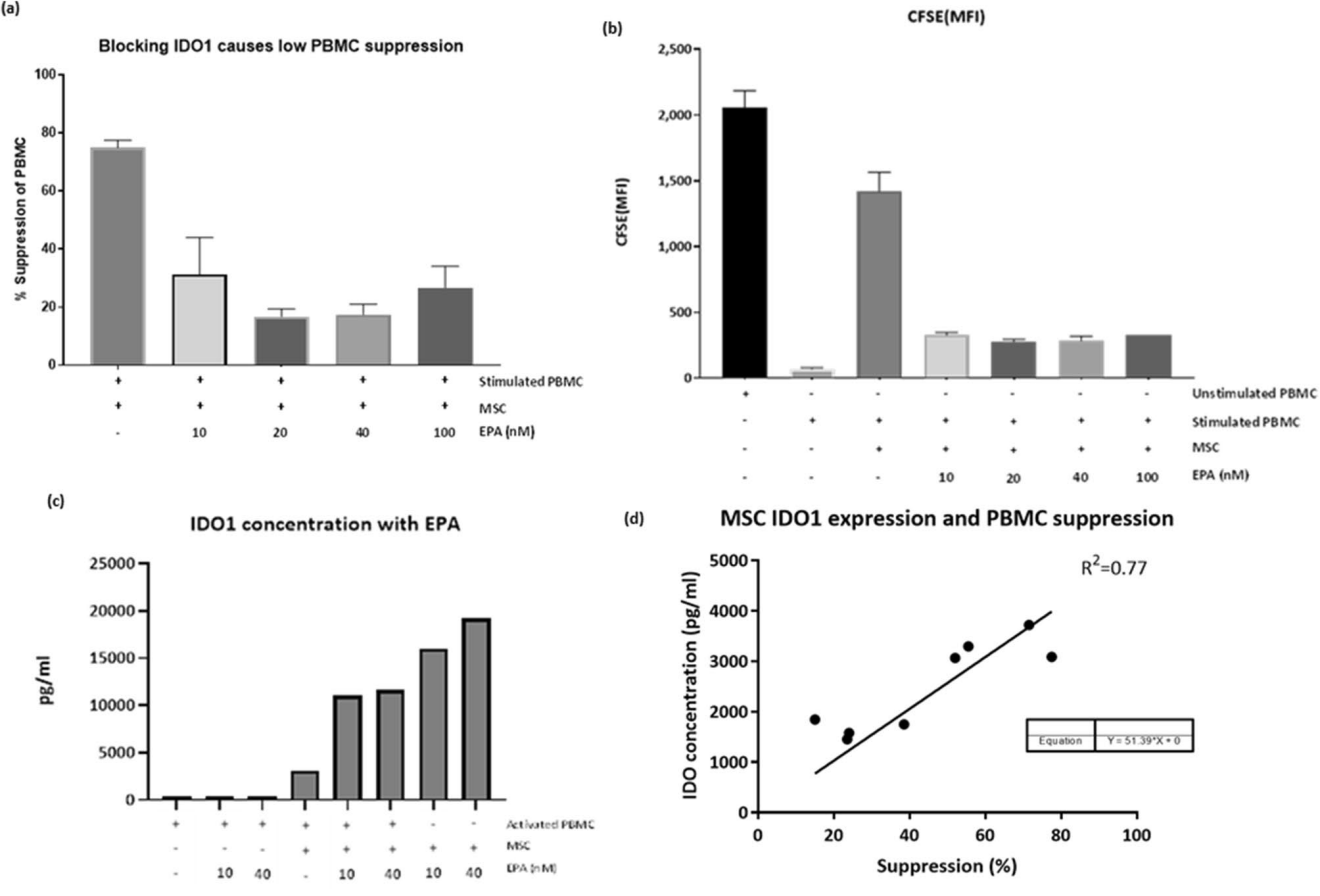
Assessment of fMSC and fPBMC whilst blocking the activity of IDO1 with (EPA) (**a**) effects of EPA on suppression PBMC (% suppression), (**b**) EPA effects on MSC mediated PBMC mean fluorescence intensity (MFI) and (**c**) effect of MSC IDO1 secretion by adding EPA to a co-culture. (**d**) Mean IDO1 secretion (n = 8) can be correlated (R^2^ = 0.77) to MSC mediated immune suppression (n = 8) and can be predicted by the equation (+) = added to culure, (−) = not added to culture.

Unstimulated PBMCs had a CFSE binding mean fluorescence intensity (MFI) of 2059 ± 249.5. Post stimulation, the PBMC MFI dropped to 70.52 ± 8.2, representing cell division. When Primed MSC were added to the culture MFI readings were closer to an unstimulated state (1421 ± 248.8) indicative of immune suppression, or impaired daughter cell division. However, when MSC were co-cultured with activated PBMC in the presence of EPA, the level of immune suppression was reduced from ~ 75 to 17% (Fig. 7a,b). Moreover, IDO1 expression by MSCs was not inhibited by EPA (Fig. 7c) rather EPA blocked IDO1 binding and inhibited the enzymatic activity necessary for T-cell activation.

To determine whether the expression of IDO1 correlated with PBMC suppression we carried out linear regression analysis of mean IDO1 levels from MSCs from males (n = 4) and females (n = 4) with their mean immunosuppression ability on activated PBMC. MSC immunosuppressive capacity significantly correlated with IDO1 expression levels (R^2^ = 0.77, Fig. 7d). Potentially providing a method for determining MSC efficacy.

## Discussion

Allogeneic donor choice in cell therapy has potential clinical implications. While choices mainly focus on donor age, MSC proliferative capacity, in-vitro potency, differentiation and cell surface biomarker characterisation, little consideration has been given to donor sex and therapeutic advantage. Haemopoietic Stem Cell research for GvHD treatment have shown benefits to sex matching, making sex a highly relevant characteristic for cellular therapy including the use of Adipose derived MSC^48^. Despite searching for improved efficacy of MSC, donor sex is often disregarded. Indeed, data is often pooled from individual donors resulting in potentially misleading claims. The sex bias associated with disease susceptibility is well recognised^30^. This study shows significant differences in the efficacy of male and female MSCs in vitro and demonstrates that female MSCs are more immunosuppressive than male MSCs.

The immune suppressive and anti-inflammatory properties of MSCs are now very well established and it is clear the disease-associated activity of various immune phenotypes is central to MSCs ability to repair injured sites. In an attempt to source efficacious MSC donors, we found Female MSC are more immunosuppressive than male MSC which we believe is a result of their response to the inflammatory microenvironment and downstream immunomodulatory protein expression which may be due to inherent hormonal differences or perhaps disparities in activation pathways.

With allogeneic MSC therapy being applied to an ever-expanding range of conditions, it is a significant problem that donor and recipient interaction and matching via sex is poorly understood and not routinely considered which probed us to investigate. Examples such as the association of the “male antigen”, H-Y when assessing male to female donor/recipient response to attenuate Graft Versus Host Disease (GVHD) are overlooked where results clearly showed a sex related antibody response^49–52^. Although not significant, we showed a trend indicating suppressive effect of fMSCs was greater on fPBMCs than mPBMCs and similarly mMSCs showed a greater suppressive effect on mPBMCs than fPBMCs (Fig. 2d). Although further research is required to determine if sex-matching MSC therapy has the potential to provide any benefits the data presented here suggests that *in-vitro*, the use of fMSCs outweighs the need for sex matching.

To function, MSCs interact with a myriad of tissues each of which are phenotypically different. As such MSC full mode of action is not yet understood. MSCs are attracted to the site of injury via inflammatory signals from activated Macrophages and T cells via mediators including TNF-α, IL-1 and IFN-y and express adhesion molecules including iCAM-1, VCAM-1 and Very Late antigen-4 (VLA-4)^53,54^. The expression of cell surface molecules both on target and administered cells is critically important when considering the cellular therapy as a exogenous injectable^43^. Their role in adhesion of immune cells and homing MSCs to the site of injury is thought to be integral in allowing MSCs to perform other functions including potent immunomodulatory paracrine effects^42,44,55–57^. However, in our in-vitro study we showed no significant differences between the expression of these markers in MSC donors despite significant differences in immunosuppressive properties. This suggests that expression of these markers when exposed to inflammation and immune suppression may be less informative of MSC potency compared to that of secreted molecules.

To gain further information on how male and female MSC respond post-acute^14^, we removed the MSCs from the direct inflammation and assessed the changes in cell surface markers. HLA-DR expression was sustained in mMSC whereas fMSC continue to highly express VCAM-1. The MHC- class II complex, HLA-DR is the master behind allorecognition and is imperative in adaptive and innate immunity and therefore transplantation. Acute immune cell responses to MSC administration involves HLA, probing us to investigate its role in MSC immunomodulation^58,59^. Serving as a call to immune action and required for antigen presentation to CD4^+^ T-cells^60^, HLA-DR plays an essential role in inflammatory diseases. Although MHC-II presentation to CD4^+^ T-cells may increase the likely-hood of MSC immunomodulation acutely, sustained expression may in turn hinder suppression^58^. Thus, mMSC sustained expression suggests it’s a potential target for immune recognition and clearance and therefore mMSC may not be functional immune suppressors.

In contrast the adhesion molecule, VCAM-1 is sustained in fMSC and may further allow MSC to adhere to immune cells thereby allowing secondary soluble mediators to take effect. The sustained expression of different cell surface markers VCAM-1 and HLA-DR, indicates potential alternate pathways to MSC response to inflammation and may prove to play a large part in overall sustained immuno modulatory variations between the sexes.

The paracrine mechanisms of MSC mediated immune modulation are well researched. IDO1, IL-1RA, PGE-2, VEGF-A IL-6, MCP-1 and HGF are all known to play roles in immune suppression and subsequent disease modification. The correlation between MSC immunosuppression and IDO1 secretion has been extensively researched and it is often reported as one of the key potency markers for MSCs^61^. IFN-y mediated IDO1 secretion from MSCs has been linked with T cell suppression as well as differentiation of monocytes into M2 macrophages^62^. Regulated mainly by the Janus Kinase and Signal Transducer and activator of transcription (JAK/STAT) pathway^63^, IFN-γ mediated IDO expression not only plays direct immunosuppressive roles but plays downstream effector secondary roles with the induction of IL-10 by anti-inflammatory Macrophages (M2) and assists in regulation of other potent immunoregulatory molecules like TNF-α stimulated gene 6 (TSG-6)^62,64^. Our data confirms these previous reports. We have shown that not only do fMSCs secrete significantly higher levels of IDO1 than mMSCs but that blocking IDO1 almost completely ablates the suppressive effect of MSCs on PBMC proliferation. This difference in IDO1 expression likely contributes towards fMSCs showing greater efficacy in modulating PBMCs proliferation.

Links between immune modulation via IDO1, IFN-γ secretion and the female hormone, oestrogen have been previously established^65,66^. It is likely that the female microenvironment intrinsically commits fMSCs toward a more suppressive phenotype. Irrespective of the mechanisms that results in IDO1 expression being higher in fMSCs than mMSCs, we showed a positive correlation between MSC expression of IDO1 and immune suppression potential. This suggests that priming MSCs and measuring IDO1 may provide a method of predicting MSC potency, putting IDO1 at the forefront of a potential potency markers for MSC related immunomodulation, a cost effective and reproducible means of determining function.

Although IDO1 represents the most significant mediator of immunosuppression, it does not work in isolation as the correlation between expression and suppression levels was less than R = 1. MSC are referred to as multi-modal or-potent and yet which molecules are required in synergy to mediate their effect is yet to be determined. Another molecule produced at higher levels in fMSC than mMSCs was Prostaglandin E-2 (PGE-2) which plays functional roles across multiple body systems including immunity, gastrointestinal, neuroendocrine and central nervous systems and its expression *in-vivo* is believed to be crucial to cell therapy^67^. Cyclooxygenase 2 (COX 2) derived PGE-2 is directly involved in immunosuppression and pain^67^. Once activated by inflammatory signals, PGE-2 from MSCs is believed to exert a range of regulatory influence on the activation status, proliferation, differentiation and function of immune cells from adaptive and innate immunity^68^. Acting by a contact or paracrine manner, PGE2 has a systemic anti-inflammatory effect of reducing TNF-α, IL-6 and vascular permeability^69^. We were able to show that female MSC secrete significantly more PGE-2 in primed MSC-S than males. Research has indicated that sex hormones play roles in controlling the presence and function of PGE-2 and may work via a feedback loop in female adipose tissue^70,71^, a possible mechanism responsible for the elevated PGE-2 response to inflammatory stimuli in fMSC and subsequent immune regulation.

Additionally, we have shown that that fMSCs expressed significantly higher levels of IL-RA than mMSC. IL-1 is an important contributor in the development of OA and other inflammatory disorders and IL-1RA is a direct antagonist to IL-1. Our data is consistent with Bessler et.al (2007) who showed that fMSC express more IL-1RA than male MSCs and that males expressed higher levels of IL-1 indicating a less suppressive phenotype^72^. Similarly, we have shown that G-CSF which plays a central role in inflammatory arthritis was increased in mMSCs compared to fMSCs. Given that blockade of the G-CSF receptor in inflammatory arthritis models has shown positive results and is now considered an efficacious target for therapy^73^ highlights that mMSCs have a less immunosuppressive phenotype than fMSCs.

The assays used, deliberately investigate how the MSC respond to inflammation relatively acutely and may not attest longevity of donor potency. However, it is widely believed that the MSC have a so-called hit and run effect from their paracrine activity on inflammatory cells, which if true indicates that fMSC should be a first port of call-in cellular therapy. The poor response to inflammatory cytokines and subsequent immune modulation seen with some mMSC may in turn be due to the regulation of Inflammatory markers like TNF-α and G-CSF and also the switching of HLA markers in response to inflammatory stimuli.

## Conclusion

Given that sex-matching in cellular therapy is gaining traction and has been shown to be of significance, donor selection for any clinical, commercial and preclinical cellular product should consider sex. Our research indicates that female adipose derived MSCs have far more potent immunomodulatory characteristics than their male counterparts and that the benefits of using fMSC as the MSC donor outweigh the potential benefits of sex-matching in MSC therapy. There is a need for further *in-vitro* and animal studies to confirm the short- and long-term advantages of sex bias, but given that MSC therapy is already happening in humans and is regarded as safe and efficacious, the groups practicing it should be mindful to consider donor/recipient sex as a means of furthering efficacy.

## Supplementary Information

Supplementary Figure S1.

Supplementary Figure S2.

Supplementary Figure S3.

Supplementary Table S1.

## Data Availability

The datasets used and/or analysed during the current study are available from the corresponding author on reasonable request.

## Abbreviations

Ab: Antibody
ABAM: Antibiotic antimycotic
CCV: Cell count & viability
CD: Cluster of differentiation
CFSE: Carboxyfluorescein diacetate succinimidyl ester
EPA: Epacadostat
FBS: Foetal bovine serum
FGFbasic: Fibroblastic growth factor
FITC: Fluorescein isothiocyanate
fMSC: Female Mesenchymal Stem Cells
G-CSF: Granulocyte colony stimulating factor
GM-CSF: Granulocyte macrophage colony stimulating factor
HGF: Hepatocyte growth factor
HLA: Human leukocyte antigen
iCAM-1: Intercellular Adhesion Molecule-1
IDO1: Indoleamine 2,3 dioxygenase
IFGR1: Interferon gamma receptor 1
IFN-y: Interferon gamma
IL-10: Interleukin 10
IL-12: Interleukin 12
IL-13: Interleukin 13
IL-15: Interleukin 15
IL-17: Interleukin 17
IL-1Ra: Interleukin 1 receptor antagonist
IL-1β: Interleukin 1 beta
IL-2: Interleukin 2
IL-4: Interleukin 4
IL-5: Interleukin 5
IL-6: Interleukin 6
IL-8: Interleukin 8
IL-9: Interleukin 9
IP-10/CXCL10: Interferon-γ-inducible protein 10/C-X-C motif chemokine 10
MCP-1/CCL-2: Monocyte chemoattractant protein-1/ C-C motif chemokine 2
MFI: Mean fluorescence intensity
MHC-I: Major histocompatibility class I
MHC-II: Major histocompatibility class II
MIP-1α/CCL3: Macrophage inflammatory protein 1 alpha/C-C motif chemokine 3
MIP-1β/CCL4: Macrophage inflammatory protein 1 beta/C-C motif chemokine 4
mMSC: Male mesenchymal stem cells
MSC: Mesenchymal stem cell
MSC-S: Mesenchymal stem cell secretome
NT5E: 5′-Nucleotidase ecto
OA: Osteoarthritis
PBMC: Peripheral blood mononuclear cells
PDGF-b: Platelet derived growth factor
PD-L1: Programmed death ligand-1
PE: Phycoerythrin
PGE-2: Prostaglandin E-2
PI: Propidium Iodide
PLT: Platelet Lysate
RANTES: Regulated upon activation, normal T cell expressed and presumably secreted
RPMI: Roswell Park Memorial Institute
SVF: Stromal vascular fraction
TGF-β1: Transforming growth factor beta-1
TLR: Toll-like receptor
TNF-α: Tumour necrosis factor alpha
VCAM-1: Vascular adhesion molecule-1
VEGF-A: Vascular endothelial growth factor
αMEM: Alpha modified eagle medium
