# Supplementary figures and images for: Immune modulation via adipose derived Mesenchymal Stem cells is driven by donor sex in vitro
Source: Sci Rep. 2021 Jun 14;11:12454. doi: 10.1038/s41598-021-91870-4 (PMC8203671; doi:10.1038/s41598-021-91870-4)

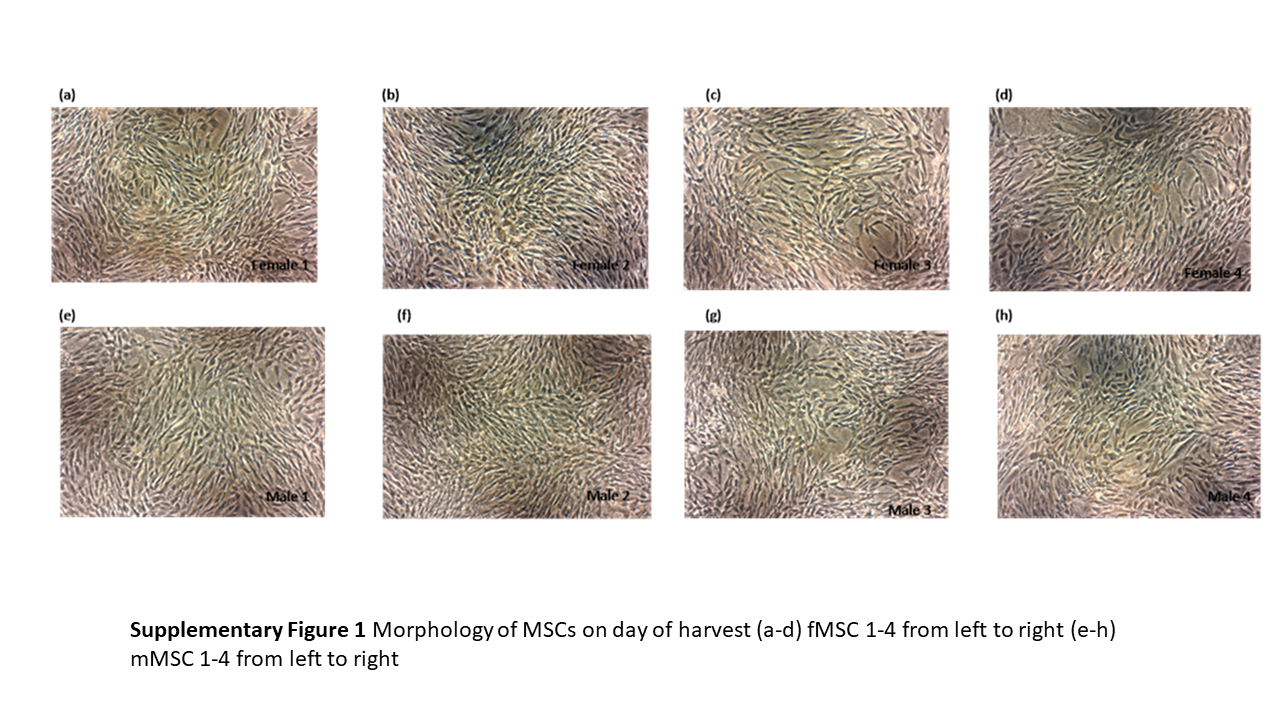

Supplement: Supplementary file 1 — Supplementary Figure S1. [file 41598_2021_91870_MOESM1_ESM.png]

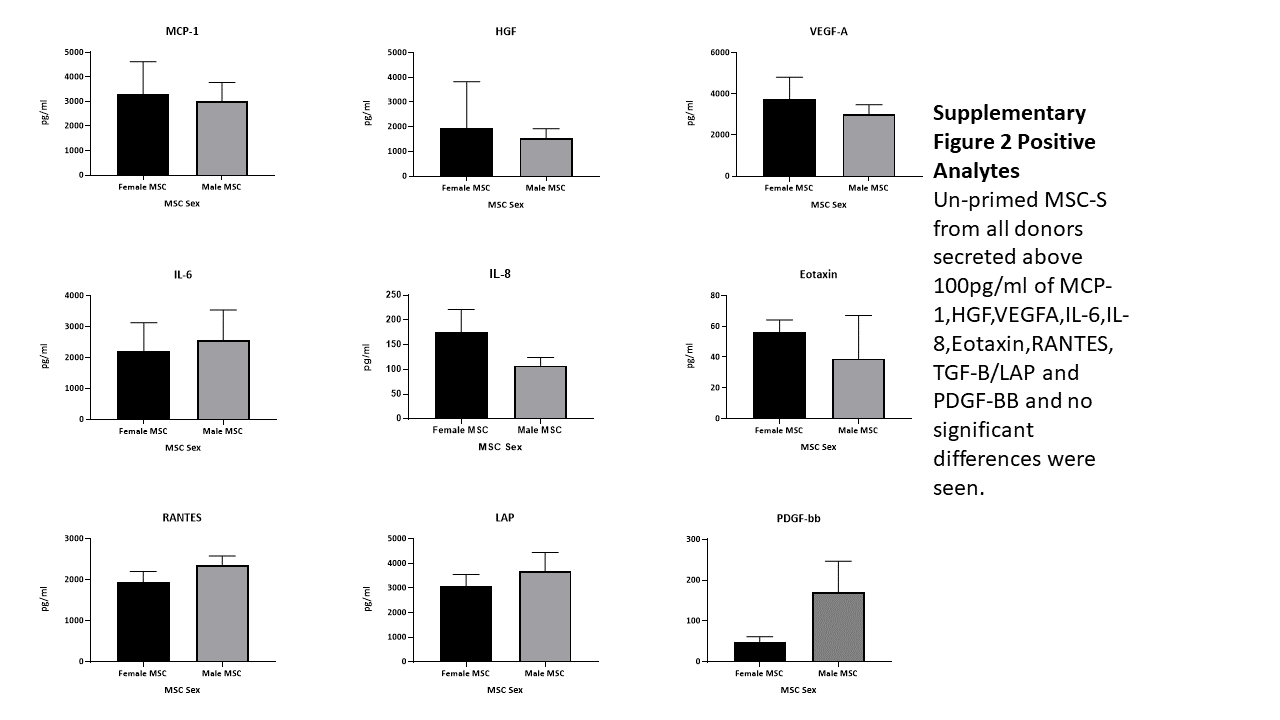

Supplement: Supplementary file 2 — Supplementary Figure S2. [file 41598_2021_91870_MOESM2_ESM.png]

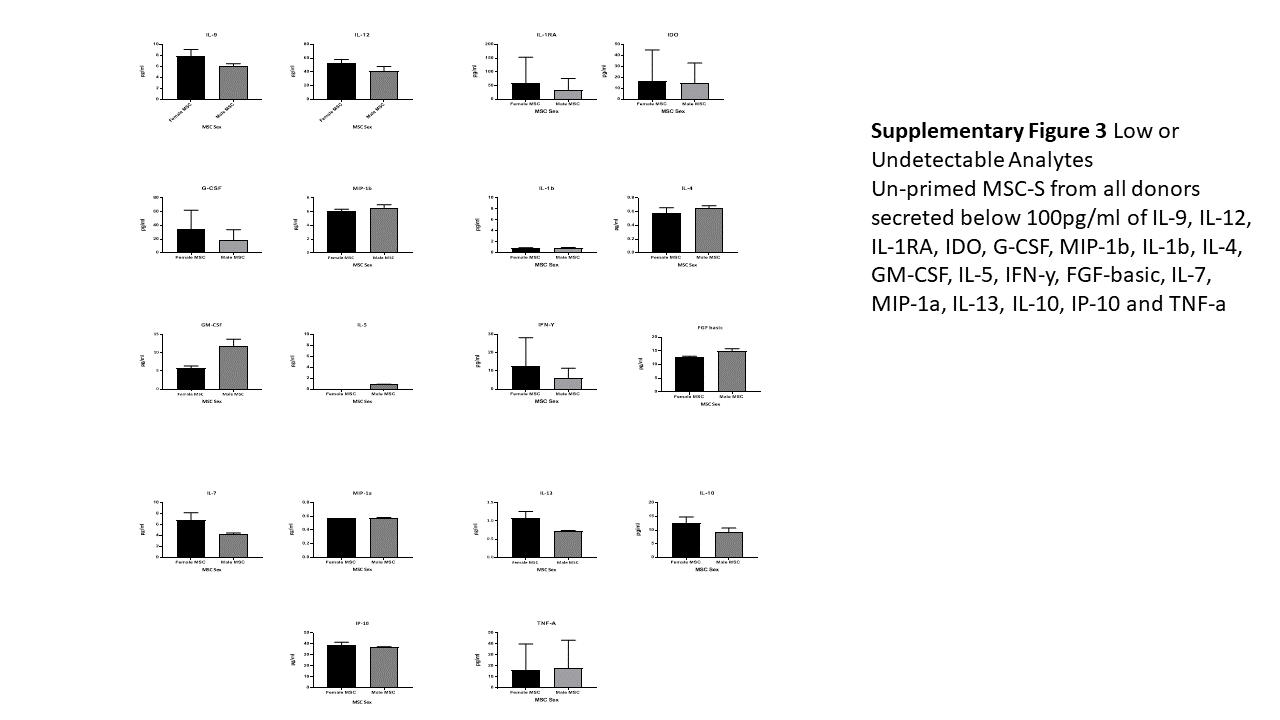

Supplement: Supplementary file 3 — Supplementary Figure S3. [file 41598_2021_91870_MOESM3_ESM.png]

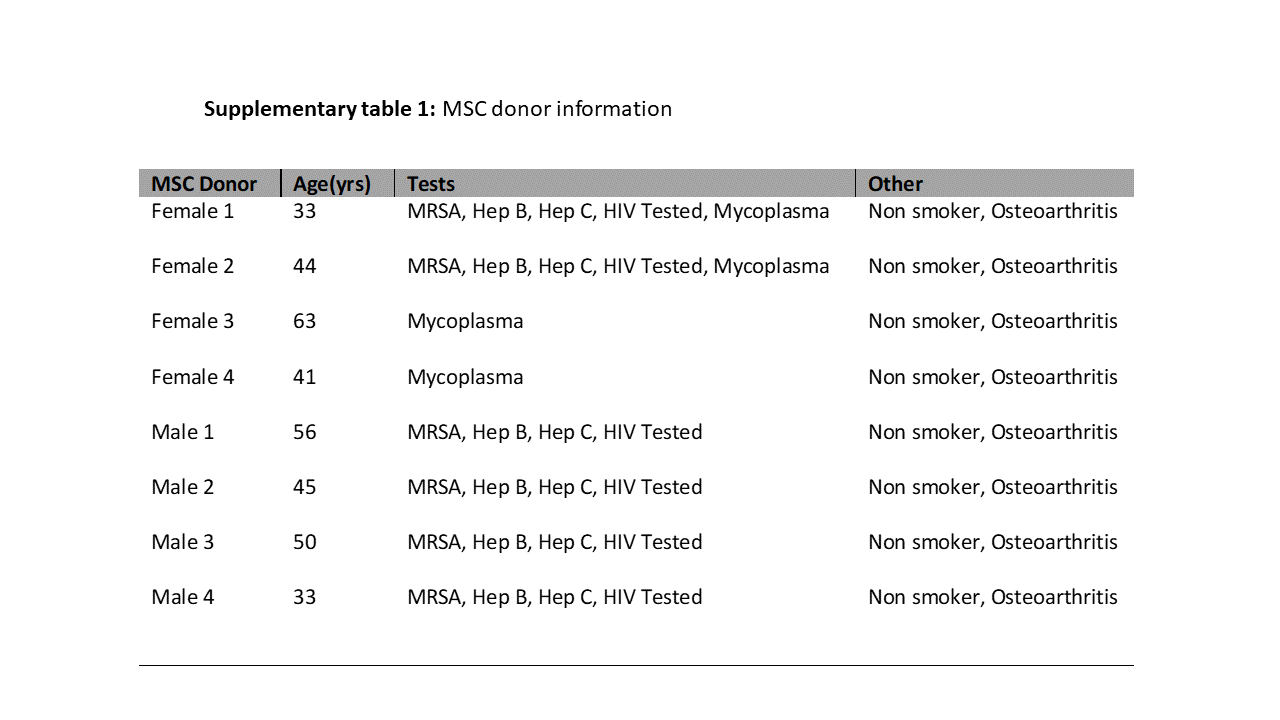

Supplement: Supplementary file 4 — Supplementary Table S1. [file 41598_2021_91870_MOESM4_ESM.png]
